# Vehicular Causation Factors and Conceptual Design Modifications to Reduce Aortic Strain in Numerically Reconstructed Real World Nearside Lateral Automotive Crashes

**DOI:** 10.1155/2015/269386

**Published:** 2015-08-31

**Authors:** Aditya Belwadi, King H. Yang

**Affiliations:** ^1^The Center for Injury Research and Prevention, The Children's Hospital of Philadelphia, 3535 Market Street, Suite 1150, PA 19104, USA; ^2^Department of Biomedical Engineering, Wayne State University, 818 W. Hancock, Detroit, MI 48201, USA

## Abstract

Aortic injury (AI) leading to disruption of the aorta is an uncommon but highly lethal consequence of trauma in modern society. Most recent estimates range from 7,500 to 8,000 cases per year from a variety of causes. It is observed that more than 80% of occupants who suffer an aortic injury die at the scene due to exsanguination into the chest cavity. It is evident that effective means of substantially improving the outcome of motor vehicle crash-induced AIs is by preventing the injury in the first place. In the current study, 16 design of computer experiments (DOCE) were carried out with varying levels of principal direction of force (PDOF), impact velocity, impact height, and impact position of the bullet vehicle combined with occupant seating positions in the case vehicle to determine the effects of these factors on aortic injury. Further, a combination of real world crash data reported in the Crash Injury Research and Engineering Network (CIREN) database, Finite Element (FE) vehicle models, and the Wayne State Human Body Model-II (WSHBM-II) indicates that *occupant seating position, impact height, and PDOF*, in that order play, a primary role in aortic injury.

## 1. Introduction

TRA and blunt aortic injury (BAI) are leading causes of death in high-speed impact trauma. Smith and Chang [1] reported on 387 cases of blunt traumatic death in vehicular crashes and found that aortic injury was second only to head injury as the leading cause of death. They also reported that nearly 85% of the victims who sustained an aortic tear died at the scene. Further, most cases of aortic injuries are accompanied by head injury, rib fractures, and/or hepatic trauma (Burkhart et al. [2]).

The mechanism of injury and the threshold for injury in these cases may be related to the particular anatomy and physiology of the aorta and the surrounding tissue. However, data from literature has shown that in lateral impacts B-pillar intrusion combined with lateral sliding of the occupant into the intruding B-pillar and associated structures are mainly responsible for aortic injury [3, 4]. Further, higher aortic strain which was seen as a primary factor for aortic tears is primarily regionalized in the peri-isthmic region, distal to the origin of the left subclavian artery [3–7].

The advent of sophisticated Finite Element (FE) computer models has in the recent years significantly aided determination of injury causation. In 2005, Shah et al. refined the first version of the human body model to develop the Wayne State Human Body Model-II (WSHBM-II) that has detailed thoracic organs including the heart, aorta, and lungs. Additional thoracic modeling, material models, and validation information can be found in Shah et al. [3]. The WSHBM has a total of 79,471 nodes and 94,484 elements with a mass of 75.6 kilograms.

## 2. Methods and Materials

To further understand the mechanism of aortic injury a cause and effect based DOCE study was performed on 16 different combinations of five design factors generated using a Latin Square method in modeFRONTIER 4.0 (ESTECO North America) [6, 8]. The reconstructions were carried out in two stages as outlined in Siegel et al. 2010. In Stage I, vehicle-to-vehicle kinematics and deformation were reconstructed from accident reports obtained from the Crash Injury Research and Engineering Network (CIREN) database (Case #7 from [4]). In Stage II, the occupant impact was considered for 16 cases. Appendices A and B describe the reconstruction process and details Case #7 for the sake of completeness.

**Figure 1:**
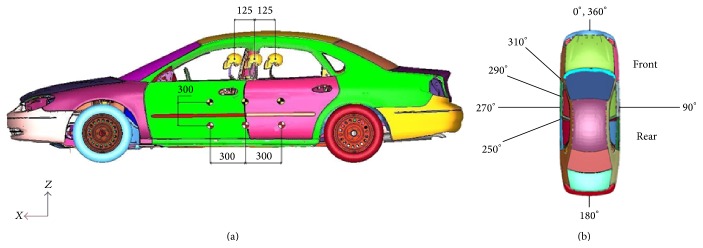
(a) Position of the impact vehicle, height of impact, and occupant seating position for Phase B simulations; (b) PDOF for Phase B simulations.

Five design factors, impact height, impact position, PDOF, and initial velocity of the bullet vehicle combined with varying occupant seating positions in the case vehicle, each with two to four levels of variations chosen from the proximity of CIREN data presented in Siegel et al. 2010 were chosen.  Table 1 lists the design factors and ranges simulated while Figures 1(a) and 1(b) graphically demonstrate these locations. Again, the vehicle kinematics time histories were used as input to the WSHBM to determine the FE model predicted risk of aorta injury.

The baseline case vehicle, a 2001 FE Ford Taurus model, similar to the struck vehicle model in the selected case was used as the target vehicle for the DOCE study. For the striking vehicle, FE models of a 2002 Dodge Caravan, which has a low bumper profile similar to a sedan, and a 2002 Ford Explorer, which has a higher bumper profile than a sedan, were used for the simulations. Impact position was chosen to be the center, 300 mm forward or 300 mm backward of the case vehicles' B-pillar. The PDOF and initial velocity were chosen to cover the range of values in previous CIREN cases. Finally, the occupant seating position selected covered the full range of for-apt range of the seat (250 mm) for a 2001 Ford Taurus with the angle of seat back at 110 degrees. That is, the occupant was positioned mid-track, 125 mm forward of mid-track, or 125 mm backward of mid-track.  Table 2 lists the outputs of DOCE using the Latin square sampling method (modeFRONTIER 4.0).

The response variables were average maximum principal strain (AMPS) and maximum pressure in the aorta. For AMPS, four adjacent elements in the region with the highest maximum principal strain were selected and averaged, while for pressure, the maximum value in the aorta obtained during the entire simulation was tabulated.

## 3. Results and Discussion

Table 2 lists the DOCE test matrix derived using a Latin square sampling from modeFRONTIER and the output variables. Maximum simulation time for each case run has been tabulated to establish a standardized time scale for comparison. Some simulations terminated earlier than the other due to “negative volume” based on LS-DYNA terminology.

It was observed from the simulation that in all runs the maximum principal strain occurred near the isthmus of the aorta, distal to the orifice of the left subclavian artery. A maximum of 32.4% strain was seen in run #5 which was a sedan impacting the B-pillar (270 degrees) at 55 km/h with the occupant seated at the B-pillar. A low of 2.5% strain was observed in run #14 which was a sedan impacting 300 mm to the left of B-pillar at an angle of 310 degrees and a velocity of 30 km/h with the occupant seated 125 mm in front of the B-pillar.

**Figure 2:**
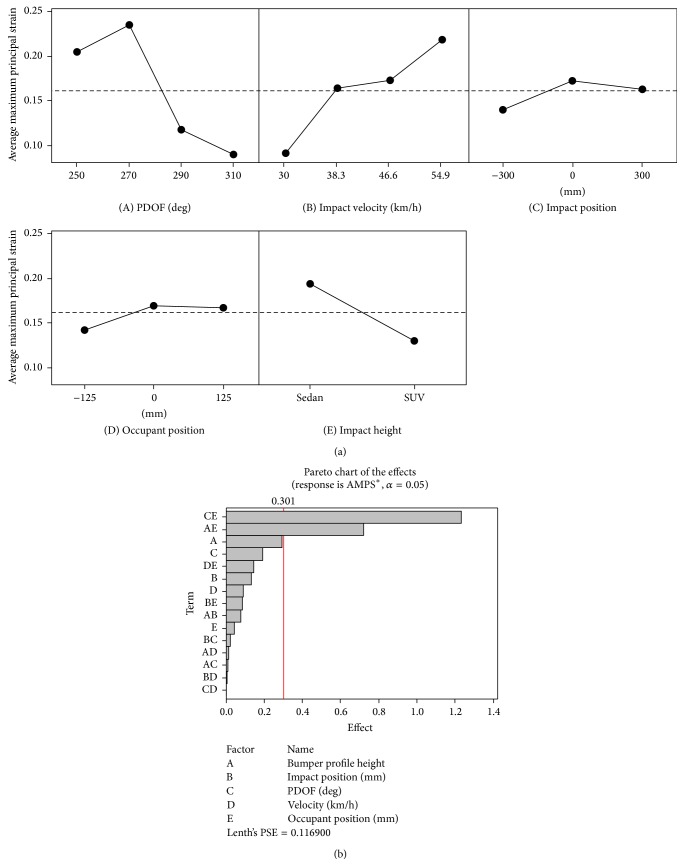
AMPS: (a) main effects chart; (b) Pareto chart of combination effects.

**Figure 3:**
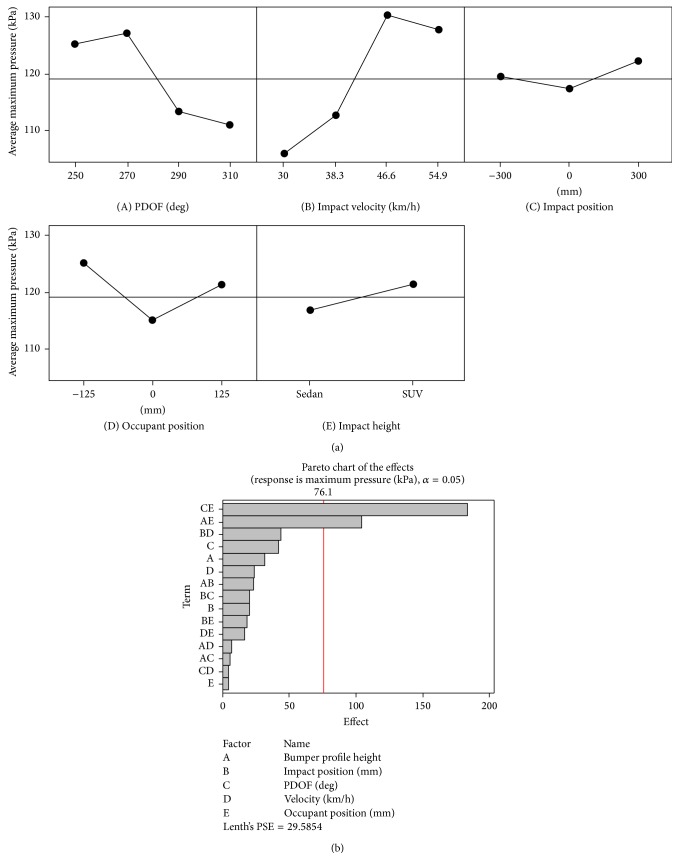
(a) Main effects chart: maximum pressure (kPa); (b) Pareto chart of combined effects.

In order to determine the critical factors, a “main effects” analysis was performed in Minitab 16.1 (Minitab Inc., PA) based on FE model predicted results listed in Table 2. Figures 2 and 3, respectively, summarize the relationship between selected design factors, AMPS in the isthmus, and peak pressure in the aorta, respectively, predicted by the WSHBM.

It is noted that a PDOF of 270 degrees resulted in the highest average AMPS (Figure 2(a)(A)) among all factors and levels studied. An increase in impact velocity had a direct correlation with the increase in maximum principal strain (Figure 2(a)(B)) while an occupant seated at the B-pillar and an impact directed to the B-pillar seemed to generate higher strain in the isthmus region (Figures 2(a)(C) and 2(a)(D)). In contrast to intuitive thinking, impacts from a Dodge caravan with a low profile bumpter generated a higher isthmus strain compared to a high profile SUV represented here by a Ford Explorer model (Figure 2(a)(E)). From the Pareto effects chart we observe that a combination of PDOF and occupant seating position followed by bumper profile height with occupant position has a significant impact on the strain generated (Figure 2(b)).

From Figure 3(a)(A), a PDOF of 270 degrees resulted in the highest aortic pressure among all four PDOFs simulated. As the impact velocity increased, the aortic pressure also increased and seemed to vary negligibly after a velocity of 46.6 km/h (Figure 3(a)(B)). In contrast to the findings for maximum principal strain, an impact position centered on the B-pillar (Figure 3(a)(C)), occupant seated at the B-pillar (Figure 3(a)(D)) generated the lowest aortic pressure, and a higher bumper profile generated a higher aortic pressure (Figure 3(a)(E)). Similar to earlier findings, the Pareto effects chart revealed a combination of PDOF and occupant seating position followed by bumper profile height with occupant position had a significant impact on the maximum pressure generated in the aorta in the 16 simulations (Figure 3(b)).

Student's *t*-test was performed to determine the level of significance for each design factor using modeFRONTIER 4.0. It was found that PDOF (*p* = 0.001) had a significant negative effect, impact velocity (*p* = 0.055) had a marginally significant positive effect, and impact height (*p* = 0.068) had a marginally significant negative effect on FE model predicted maximum principal strain. The impact position (*p* = 0.295) and occupant position (*p* = 0.304) did not significantly affect the FE model predicted maximum principal strain. In terms of FE model predicted peak aortic pressure, impact velocity (*p* = 0.002) had a significant positive effect while PDOF (*p* = 0.028) had a significant negative effect. Other factors, occupant position (*p* = 0.185), impact height (*p* = 0.283), and impact position (*p* = 0.475), did not significantly affect the FE model predicted aortic pressure.

Bass et al. [9] reported a 50% risk of tear to the aorta at 120 kPa for occupants 68 years of age. Further, Shah et al. in 2006 [10] tested eight cruciate shaped cadaveric aortas until failure utilizing a biaxial fixture and reported an average longitudinal failure strain of 22.1%. They defined failure to be a complete tear of all three layers of the aorta (tunica intima, tunica media, and tunica adventitia). Utilizing strain (0.221) and pressure (120 kPa) as thresholds for aortic failure, Table 3 reports the average AMPS and average maximum pressure along with their significance for each factor analyzed separately for failure based on strain and pressure criterion.

It is seen from Table 3 that for a strain based criterion the number of runs with failure was significant for AMPS while for a pressure based criterion, the number of failure runs was significant for maximum pressure. There was no correlation found between aortic failure with strain and pressure combined. This was also supported by data from Table 2 where no correlation was found between times of occurrence of maximum AMPS and maximum pressure in the aorta for a particular run.

Although there was no significant difference in impact velocity, PDOF, or maximum pressure it is interesting to note that the runs with aortic failure had a bullet vehicle with a low bumper profile (sedan). It was observed that in runs with lower bumper profile the armrest gets pushed into the thorax while it is completely missed with a higher bumper profile. Further, it was also seen that there was a mass difference of 488.5 kilograms between the Dodge Caravan (2028.1 kgs) and the Ford Explorer (1539.6 kgs) FE models. The difference in momentum between the two impacts might have had an effect on the intrusion pattern. A one-way ANOVA performed between the two FE models for average maximum principal strain (*p* = 0.136) and maximum pressure in the aorta (*p* = 0.58) was not significant.

Several limitations of the current study are noted. Even though the vehicle models were accurately scaled to match the size and weight of the case vehicle, the stiffness and interior compartment details were not compensated. It is also important to observe that measured external deformation may not correspond to similar occupant compartment intrusion and contact force due to differences in elastic modulus of various interior components. This problem is exacerbated by the fact that deformation profiles are measured at individual points on the external surface leading to variations in actual and simulated profiles.

## 4. Conclusions

Sixteen DOCE runs were carried out using FE vehicle models and the second version of the Wayne State Human Body Model. In simulated nearside left lateral crashes, peak average maximum principal strain primarily occurred in the isthmus of the aorta, distal to the orifice of the left subclavian artery. Results of design of computer experiments concluded that* occupant seating position, bumper profile height, and PDOF of impact,* in that order, play a crucial role in the generation of strain and pressure in the aorta, a potential injury mechanism responsible for traumatic rupture of the aorta in automobile crashes.

## Figures and Tables

**Figure 4:**
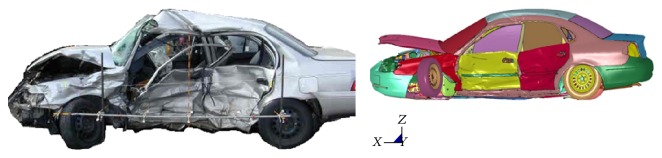
Vehicle deformation comparison of simulated FE vehicle against actual vehicle,* CASE 7*.

**Figure 5 fig5:**
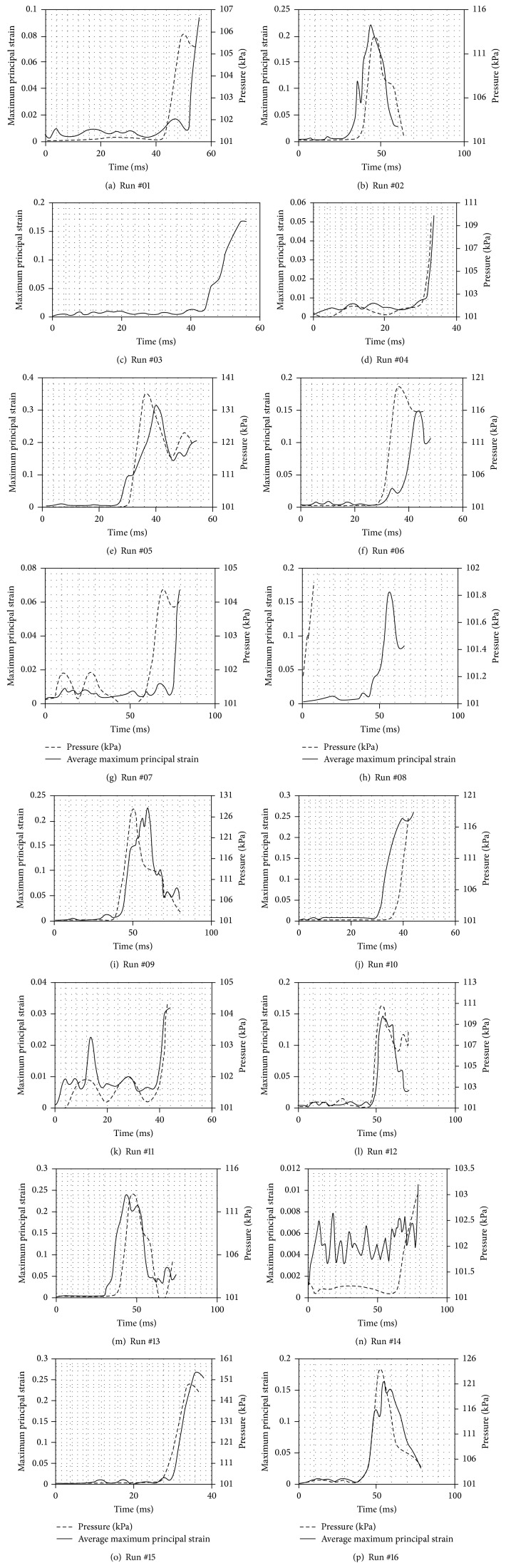
Average maximum principal strain in the isthmus and pressure: runs #01 through #08. Average maximum principal strain in the isthmus and pressure: runs #09 through #16.

**Table 1:** Range of values for the five design factors chosen for the DOCE study.

Number	Design factor	Range			
	Striking vehicle				
1	Bumper profile	Low	High	—	—
2	Impact position (mm)^*∗*^	−300	0	+300	—
3	PDOF (degrees)	250	270	290	310
4	Initial velocity (km/h)	30	38.3	46.6	54.9
5	Occupant position (mm)^*∗*^	−125	0	+125	—

^*∗*^ *Note*. Impact position and occupant position are determined from the center of the case vehicles' B-pillar.

**Table 2:** Latin square sampling for DOCE and output response variables; average maximum principal strain (AMPS) and maximum pressure in the aorta.

Run #	Bumper profile height	Impact position (mm)	PDOF (degrees)	Velocity (km/h)	Occupant position (mm)	Maximum simulation time (ms)	AMPS^*∗*^	Time at AMPS (ms)	Maximum pressure (kPa)	Time at maximum pressure (ms)
1	Low	−300	290	30	+125	56	0.1180	55	105.8	50
2	Low	−300	270	38.3	0	65	0.2240	44	113.5	46
3	High	−300	250	46.6	−125	54	0.1650	54	148.0	48
4	High	−300	310	54.9	0	33	0.0540	33	109.0	33
5	Low	0	270	54.9	0	52	0.3240	40	135.0	36
6	Low	0	290	46.6	+125	46	0.1580	44	119.6	36
7	High	0	310	30	0	78	0.0675	78	104.4	70
8	High	0	250	38.3	−125	64	0.1650	56	120.0	50
9	Low	0	310	46.6	−125	80	0.2100	60	127.6	50
10	Low	0	250	54.9	0	44	0.2580	43	117.7	42
11	High	0	290	38.3	0	44	0.0330	43	104.3	42
12	High	0	270	30	+125	70	0.1520	54	110.8	54
13	Low	+300	250	38.3	0	72	0.2300	44	113.2	48
14	Low	+300	310	30	−125	80	0.0250	78	103.0	78
15	High	+300	270	54.9	+125	36	0.2350	34	149.0	34
16	High	+300	290	46.6	0	76	0.1600	54	123.7	52

^*∗*^Average maximum principal strain (%) = lower surface average maximum tensile principal strain in the longitudinal axis of the aorta.

^*∗*^AMPS and maximum strain curves for each run are presented in Figure 5.

**Table 3:** Failure versus nonfailure values of AMPS and maximum pressure in the aorta assessed based on failure criterion.

	Failure runs	Nonfailure runs	Significance (*p*)
Failure criterion = 22.1% strain [3]			
Average AMPS	0.247 ± 0.041	0.109 ± 0.058	0.001
Maximum pressure (kPa)	126.00 ± 14.15	114.86 ± 13.82	0.155

Failure criterion = 120 kPa pressure [9]			
Average AMPS	0.203 ± 0.061	0.129 ± 0.091	0.076
Maximum pressure (kPa)	131.85 ± 12.51	109.08 ± 5.017	0.003

## Appendices

### A. FE Reconstruction Methodology

For the case and bullet vehicles, a 2001 Ford Taurus and 2002 Dodge Caravan FE models, respectively, downloaded from the National Crash Analysis Center (NCAC) website were scaled to match with the overall dimensions such as wheelbase, width, and height. In addition, the vehicle mass was adjusted by removing a few components, such as the rear bumper, which would typically not be involved in left lateral or frontal crashes. The driver's weight was compensated for by adding a lumped mass (from the case data) to the center of gravity of the driver's seat. Care was taken to ensure that the overall center of gravity and total mass were not altered. Similarly, the striking vehicle was modeled, and the two vehicles were positioned as suggested by the crash investigation data. Initial velocity was applied to the striking vehicle as a vector component defined by the PDOF. The total simulation time was assigned to ensure maximum deformations were reached (elastic-plastic). All simulations were modeled using Hypermesh 9.0 (Altair Corporation, Troy, MI) as the preprocessor, a Massively Parallel Platform (MPP) version of LS-DYNA 970 on a four-node cluster (two processors per node) as the solver and LS-PrePost 2.4 (LSTC Corporation, Livermore, CA) as the postprocessor. Structural deformation depths obtained in the simulations were compared with the deformation profile C1 to C6 reported as per SAEJ2433 in the CIREN data (Figure 4 [Fig fig5]).

A local coordinate system was established on the struck case vehicle to obtain deformation in the local axis. For the case vehicles, the driver side structures (including the front and rear doorframe, door armrest, and left B-pillar nodes) were grouped and their motions were recorded in separate binary interface files. These interface files were used as inputs for Stage II simulations.

In Stage II, the interface files which consist of nodal kinematic histories and the submodel (left door structures) of the case vehicles' structures that might interact with the occupant were used as inputs to the WSHBM occupant model. The WSHBM was positioned as per the range of values for the DOCE setup defined in Table 1. A contact interface was created between the interior structures of the vehicle interior submodel and the occupant model. Simulation output provided the overall occupant kinematics at the time of the peak vehicle deformation and the average maximum principal strain (AMPS) and maximum pressure in the aorta predicted by the occupant model.

### B. Case #7 Description

This case involved a 34-year-old African-American male driver (weight = 83 kgs and height = 1630 mm) of a 1993 Toyota Corolla (weight = 1085 kgs) struck broadside by a 1996 Dodge Caravan (weight = 1085 kgs). The subject was utilizing the three-point belt system and the frontal air bag deployed at the time of impact. The patient sustained fatal injuries including a 30 mm transverse laceration of the aortic isthmus on the posterior right side of the isthmus, located 35 mm distal to the left subclavian artery orifice. There was an associated aortic dissection and mediastinal hemorrhage. There was a second fatal injury involving a basilar skull fracture of the “hinge” type with atlantooccipital dislocation. The driver was dead at the scene. The Delta-V on impact for the case vehicle calculated by WinSmash was 59 kph with an impact at a PDOF of 280 degrees.

## References

[1] Smith R. S., Chang F. C. (1986). Traumatic rupture of the aorta: still a lethal injury. *The American Journal of Surgery*.

[2] Burkhart H. M., Gomez G. A., Jacobson L. E., Pless J. E., Broadie T. A. (2001). Fatal blunt aortic injuries: a review of 242 autopsy cases. *Journal of Trauma—Injury, Infection and Critical Care*.

[3] Shah C. S., Hardy W. N., Yang K. H., Van Ee C. A., Morgan R. M., Digges K. H. Investigation of the traumatic rupture of the aorta (TRA) by simulating real-world accidents.

[4] Siegel J. H., Belwadi A., Smith J. A., Shah C., Yang K. (2010). Analysis of the mechanism of lateral impact aortic isthmus disruption in real-life motor vehicle crashes using a computer-based finite element numeric model: with simulation of prevention strategies. *Journal of Trauma—Injury, Infection and Critical Care*.

[5] Shah C. S., Maddali M., Mungikar S. A. Analysis of a real-world crash using finite element modeling to examine traumatic rupture of the aorta.

[6] Belwadi A., Siegel J. H., Singh A., Smith J. A., Yang K. H., King A. I. (2012). Finite element aortic injury reconstruction of near side lateral impacts using real world crash data. *Journal of Biomechanical Engineering*.

[7] Hardy W. N., Shah C. S., Mason M. J. (2008). Mechanisms of traumatic rupture of the aorta and associated peri-isthmic motion and deformation. *Stapp Car Crash Journal*.

[8] Bammel S. E., Rothstein J. (1975). The number of 9×9 latin squares. *Discrete Mathematics*.

[9] Bass C. R., Darvish K., Bush B. (2001). Material properties for modeling traumatic aortic rupture. *Stapp Car Crash Journal*.

[10] Shah C. S., Hardy W. N., Mason M. J. (2006). Dynamic biaxial tissue properties of the human cadaver aorta. *Stapp Car Crash Journal*.

